# Change in treatment burden among people with multimorbidity: a follow-up survey

**DOI:** 10.3399/BJGP.2022.0103

**Published:** 2022-09-06

**Authors:** Hilda O Hounkpatin, Paul Roderick, Scott Harris, James E Morris, Dianna Smith, Bronagh Walsh, Helen C Roberts, Hajira Dambha-Miller, Qian Yue Tan, Forbes Watson, Simon DS Fraser

**Affiliations:** National Institute for Health and Care Research (NIHR) clinical lecturer in primary care and GP;; National Institute for Health and Care Research (NIHR) clinical lecturer in primary care and GP;; National Institute for Health and Care Research (NIHR) clinical lecturer in primary care and GP;; National Institute for Health and Care Research (NIHR) clinical lecturer in primary care and GP;; Geography and Environmental Science, University of Southampton, Southampton.; NIHR academic clinical fellow in geriatric medicine, Academic Geriatric Medicine, Human Development and Health, University of Southampton, Southampton.; Academic Geriatric Medicine, Human Development and Health, University of Southampton, Southampton; Medicine for Older People, University Hospitals Southampton, Southampton.; National Institute for Health and Care Research (NIHR) clinical lecturer in primary care and GP;; NIHR academic clinical fellow in geriatric medicine, Academic Geriatric Medicine, Human Development and Health, University of Southampton, Southampton.; NHS Dorset Clinical Commissioning Group, Dorset.; School of Primary Care, Population Science and Medical Education, Faculty of Medicine, University of Southampton, Southampton.

**Keywords:** epidemiology, general practice, multimorbidity, patient-centred care, self-management

## Abstract

**Background:**

Treatment burden is the effort required of patients to look after their health and the impact this has on their functioning and wellbeing. Little is known about change in treatment burden over time for people with multimorbidity.

**Aim:**

To quantify change in treatment burden, determine factors associated with this change, and evaluate a revised single-item measure for high treatment burden in older adults with multimorbidity.

**Design and setting:**

A 2.5-year follow-up of a cross-sectional postal survey via six general practices in Dorset, England.

**Method:**

GP practices identified participants of the baseline survey. Data on treatment burden (measured using the Multimorbidity Treatment Burden Questionnaire; MTBQ), sociodemographics, clinical variables, health literacy, and financial resource were collected. Change in treatment burden was described, and associations assessed using regression models. Diagnostic test performance metrics evaluated the revised single-item measure relative to the MTBQ.

**Results:**

In total, 300 participants were recruited (77.3% response rate). Overall, there was a mean increase of 2.6 (standard deviation 11.2) points in treatment burden global score. Ninety-eight (32.7%) and 53 (17.7%) participants experienced an increase and decrease, respectively, in treatment burden category. An increase in treatment burden was associated with having >5 long-term conditions (adjusted β 8.26, 95% confidence interval [CI] = 4.20 to 12.32) and living >10 minutes (versus ≤10 minutes) from the GP (adjusted β 3.88, 95% CI = 1.32 to 6.43), particularly for participants with limited health literacy (mean difference: adjusted β 9.59, 95% CI = 2.17 to 17.00). The single-item measure performed moderately (sensitivity 55.7%; specificity 92.4%.

**Conclusion:**

Treatment burden changes over time. Improving access to primary care, particularly for those living further away from services, and enhancing health literacy may mitigate increases in burden.

## INTRODUCTION

Treatment burden is the effort required of patients to look after their health and the impact this has on their functioning and wellbeing.^1^^–^4 Taking and managing multiple medications, organising healthcare appointments, monitoring health, performing self-care, and modifying lifestyle contribute to this workload.^3^ This workload may be substantially increased for people with multiple long-term conditions (LTCs), potentially outweighing their capacity to manage their health.^5^ High treatment burden may be associated with poor adherence to treatment, poorer clinical outcomes, and healthcare inefficiency.^6^^–^9 Given the increasing prevalence of multimorbidity, it is important for health services to be organised in ways that reduce treatment burden and improve quality of life for patients and carers.^10^^,^^11^

Recently, changes to healthcare delivery have been implemented to improve services for patients.^12^^–^14 Following a major review of the NHS, integrated care systems are endeavouring to align primary and specialist care, physical and mental health services, and health with social care.^15^ Treatment burden may therefore have decreased over time as a result of improved access to health care for some patients, but, to the authors’ knowledge, no population-level quantitative studies in the UK have assessed changes in treatment burden, making it impossible to assess the impact of health system change on this patient-centred metric. Furthermore, it is not clear which patient groups are more likely to experience an increase or decrease in treatment burden. Although previous studies have identified patient characteristics associated with high treatment burden,^16^^,^^17^ it is not clear whether these factors also influence change in treatment burden. This understanding could help predict patient trajectories and the planning of future interventions and healthcare delivery, to better meet patient needs and reduce avoidable treatment burden.

At the individual (patient–clinician) level, reductions in treatment burden may be achieved by discussing with patients how best to optimise care.^10^^,^^18^^,^^19^ However, there is currently no swift and accurate method to assess treatment burden during clinical encounters. The authors have previously explored the performance of a novel single-item measure of patient-perceived treatment burden and found that it performed moderately, suggesting further development was needed before such a measure can be adopted in practice.^17^

**Table:** How this fits in

The extent to which treatment burden changes over time and which groups of people are likely to experience increases or decreases in treatment burden is not known. This study identified that a third of older adults with multimorbidity experienced an increase in treatment burden category (9% moved to the ‘high’ treatment burden category) over 2.5 years, and that living >10 minutes away from their GP, particularly for those with limited health literacy, was associated with an increase in treatment burden. Improving patient access to primary care services and enhancing health literacy may help to mitigate increases in treatment burden. The authors’ revised single-item measure performed moderately, suggesting a brief measure of treatment burden consisting of more than one item may be required for use in practice.

This study therefore aimed to quantify change in treatment burden over time and determine factors associated with this change in older adults with multimorbidity. The single-item treatment burden measure was also revised and its performance evaluated.

## METHOD

### Survey design and sample

This was a 2.5-year follow-up study of a cross-sectional survey on treatment burden in 835 older adults (aged ≥55 years) with multimorbidity in Dorset, England.^17^ The follow-up survey was planned before the COVID-19 pandemic and implementation of integrated care systems in Dorset. The follow-up postal survey, conducted between August and December 2021, was sent to people who had participated in the baseline survey and consented (at baseline) to receiving a follow-up survey. Inclusion and exclusion criteria for the baseline survey are presented in Box 1.

**Box 1.** Inclusion and exclusion criteria for baseline survey

**Inclusion criteria** Aged ≥55 years Living at home with ≥3 specified long-term conditions (defined using the Quality and Outcomes Framework clinical code clusters and Read codes): atrial fibrillation, coronary heart disease, heart failure, hypertension, peripheral arterial disease, stroke or transient ischaemic attack, diabetes, asthma, chronic obstructive pulmonary disease, depression, chronic kidney disease, epilepsy, osteoporosis, rheumatoid arthritis, Parkinson’s disease, multiple sclerosis, inflammatory bowel disease, coeliac disease, and osteoarthritis. Reasons for choosing these conditions are discussed elsewhere^17^ **Exclusion criteria** Patients who were living in a care home; receiving palliative care; had a serious mental health diagnosis (for example, psychosis, schizophrenia, or bipolar disorder), dementia, or active cancer (recorded in the past 3 years); expressed a wish not to participate in research; lacked mental capacity to participate in the study; or were deemed (by healthcare professionals at the GP practice with sufficient knowledge of the patient) unsuitable to receive the survey.^17^^,^^20^

### Follow-up recruitment, invitation, and response

All eight GP practices participating in the baseline study were asked to identify patients who responded to the baseline survey and consented to receiving the follow-up survey. Six of the eight practices were able to participate. Practices manually screened these participants for the same exclusion criteria as at baseline, excluding those who now met these criteria. Practices then posted survey packs to eligible patients. Recruitment and invitation processes were similar to the baseline survey, which are described in full elsewhere.^17^^,^^20^

### Data collection

#### Treatment burden outcome measure

Treatment burden was measured using the 10-item Multimorbidity Treatment Burden Questionnaire (MTBQ).^21^ The MTBQ was validated in a similar population and is a concise, easy-to-use measure that asks questions about difficulty with medication, healthcare appointments, and lifestyle changes. Possible responses to each item on the MTBQ are: 0 (not difficult/does not apply); 1 (a little difficult); 2 (quite difficult); 3 (very difficult); and 4 (extremely difficult). For patients completing ≥5 items, a global score is then calculated by multiplying the average item score by 25 to yield a score ranging from 0 to 100. Treatment burden was also categorised to none (global score of 0), low (>0 and <10), medium (≥10 and <22), or high (≥22).

A revised single-item tool that asked: ‘Have you felt overstretched by everything you’ve had to do to manage your health in the last month (for example, taking medicines, getting prescriptions, attending appointments)?’ to which participants could respond ‘yes’ or ‘no’ was also included. This measure emerged from focus group discussions with patient and public contributors with lived experiences of multimorbidity (Box 2).^17^

**Box 2.** Development of revised single-item treatment burden measure

Original question: *‘Please consider the overall effort of looking after your health. On a scale of 0–10, where 0 is no effort and 10 is the highest effort you can imagine, how would you rate the amount of effort you have to put in to manage your health conditions?’* The authors explored ways in which the initial treatment burden question based on a number-line could be improved using a 90-minute workshop involving two patient and public contributor members of the study’s patient and public involvement (PPI) team and seven other PPI representatives from the National Institute for Health Research Applied Research Collaboration for Wessex ( *n* = 9 in total). This involved the following: open discussions about how best a single-item question could be worded to reflect the patient perspective of treatment burden, with guiding questions; the next stage was a virtual iteration of the question; the group were then sent three potential questions and asked to rank them by preference; and the authors summed the rankings and selected the question with highest ranking.

#### Independent variables

Data on number of prescribed regular medications and dosing frequency, specific LTCs (based on survey inclusion criteria), and mode and travel time to healthcare services were collected. Participants were also asked to specify whether they had attended hospital in an emergency (to accident and emergency and/or stayed overnight), hospital outpatient appointment, or GP appointment within the previous 6 months, and, if so, the number of each. Perceived level of difficulty in meeting financial costs of health care was assessed on a five-point Likert scale ranging from ‘not difficult/not applicable’ to ‘extremely difficult’. Perceived frequency of needing help to read health-related written material was assessed using the Single Item Literacy Screener, measured on a five-point Likert scale and categorised into ‘not limited’ (‘never’ or ‘rarely’) or ‘limited’ (‘sometimes’, ‘often’, or ‘always’).^22^

Sociodemographic data included age (as a continuous variable), sex (male or female), marital status (married or in a civil partnership, single [never married or in a civil partnership], divorced or dissolved civil partnership, or widowed). Home ownership (homeowner or non-homeowner) and education level (in three categories) were also included as indicators of socioeconomic status.

Survey data underwent manual database input by the first author with careful rechecking to minimise risk of input errors.

### Statistical analysis

The maximum available sample was, to some extent, determined by the sample size of the baseline survey.^17^ A sample size calculation using nQuery (version 7.0) indicated that 154 patients were needed to detect the minimum possible change in treatment burden as measured by the MTBQ (one point in the MTBQ raw score).^20^

Descriptive statistics compared the number, proportions, and characteristics of participants reporting ‘none’, ‘low’, ‘medium’, and ‘high’ levels of treatment burden (at follow-up) and change in global score and treatment burden categories over time. Characteristics of participants who experienced an increase in treatment burden category and those who did not were compared using *t*-tests (for normally distributed variables) and χ^2^-tests (for categorical variables). Similar comparisons were made for those who experienced a decrease in treatment burden category. Univariable and multivariable mixed regression models (including GP practice [as a random effect]) assessed associations with change in treatment burden and baseline characteristics of participants. The authors first fitted a linear mixed model regressing change in treatment burden (as a continuous outcome) on baseline patient characteristics and adjusted for clustering at GP practice (as a random effect). This model was repeated additionally adjusting for potential confounders (age, sex, and marital status), treatment burden category at baseline, and any variables that were found to be significant in univariable models. A limited number of potential two-way interactions were also considered between travel time for health care and home ownership, financial difficulty, and health literacy, as effects may vary across these groups. These potential interactions were considered one at a time, as additions to the otherwise final, fully adjusted model.

Generalised logistic mixed regression models were then fitted to assess associations with the binary outcomes of an increase (versus no change or a decrease) or decrease (versus no change or an increase) in treatment burden. These models included GP practice as a random effect and additional adjusting variables in a similar style to the linear mixed models.

Sensitivity, specificity, positive predictive value, negative predictive value, and positive and negative likelihood ratios were calculated to evaluate the single-item tool relative to the MTBQ. A receiver operating characteristic curve was also plotted and the area under the curve (AUC) estimated to evaluate the ability of the single-item measure to discriminate between high- and non-high-treatment burden. Analyses were conducted in Stata (version 15).

## RESULTS

### Study population

**Figure 1.**
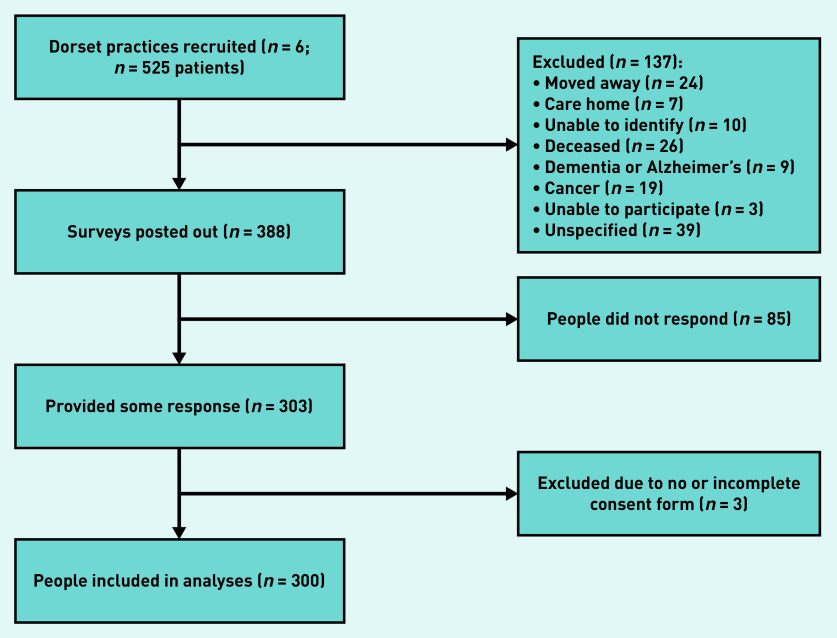
*Survey flowchart: recruitment, invitation, and response.*

A total of 525 potentially eligible patients were identified and practices posted out 388 survey packs, after excluding 137 patients who met exclusion criteria. The survey response rate was 77.3%, with 300 participants returning completed surveys and consent forms (Figure 1).

The mean age was 74.5 (standard deviation [SD] 8.0) years (Table 1). Most were of White ethnicity ( *n* = 298/299, 99.7%), female ( *n* = 171/300, 57.0%), retired ( *n* = 265/300, 88.3%), married or in a partnership ( *n* = 189/300, 63.0%), and a homeowner ( *n* = 250/298, 83.9%). Many participants ( *n* = 130, 43.3%) reported medium ( *n* = 84, 28.0%) or high ( *n* = 46, 15.3%) treatment burden at baseline. Characteristics of study participants were similar to those of the invited sample (Table 1 and Supplementary Table S1).

**Table 1.** Baseline characteristics of follow-up survey responders (*N* = 300)

**Characteristic**	***n* (%)^a^**
**Age, years, mean (SD)**	74.5 (8.0)

**Age category, years (*n*= 298)**	
55–59	14 (4.7)
60–64	17 (5.7)
65–69	45 (15.1)
70–74	77 (25.8)
75–79	66 (22.1)
80–84	44 (14.8)
85–89	28 (9.4)
90–94	7 (2.3)

**Sex (*n*= 300)**	
Male	129 (42.9)
Female	171 (56.8)

**Education level (*n*= 291)**	
NVQ4/NVQ5/Degree	80 (27.5)
NVQ3/GCE A Level/4NVQ2/GCE O Level/5NVQ1/CSE other grade	92 (31.6)
No qualification	118 (40.5)
Other	1 (0.3)

**Ethnicity (*n*= 299)**	
White	298 (99.7)
Other	1 (0.3)

**Marital status (*n*= 300)**	
Married or in a partnership	189 (63.0)
Widowed	69 (23.0)
Divorced or dissolved partnership	31 (10.3)
Single	11 (3.7)

**Living situation (*n*= 299)**	
Cohabiting	204 (68.2)
Lives alone	95 (31.8)

**Home ownership (*n*= 298)**	
Homeowner	250 (83.9)
Non-homeowner	48 (16.1)

**Employment status (*n*= 300)**	
Retired	265 (88.3)
Employed	23 (7.7)
Unemployed	6 (2.0)
Other	6 (2.0)

**Smoking status (*n*= 300)**	
Current smoker	13 (4.3)
Ex-smoker	153 (51.0)
Never smoked	134 (44.7)

**Long-term conditions (*n*= 300)**	
0	6 (2.0)
1	27 (9.0)
2	89 (29.7)
3	94 (31.3)
4	50 (16.7)
5	19 (6.3)
≥6	15 (5.0)

**Medications prescribed (*n*= 298)**	
0–3	46 (15.4)
4–6	108 (36.2)
7–9	90 (30.2)
10–14	40 (13.4)
≥15	14 (4.7)

**Treatment burden category (*n*= 300)**	
High	46 (15.3)
Medium	84 (28.0)
Low	86 (28.7)
None	84 (28.0)

**Health literacy (*n*= 299)**	
Never	210 (70.2)
Rarely	53 (17.7)
Sometimes	23 (7.7)
Often	8 (2.7)
Always	5 (1.7)

**Financial difficulty with health care (*n*= 297)**	
Not difficult or not applicable	236 (79.5)
A little	44 (14.8)
Quite	10 (3.4)
Very	7 (2.4)
Extreme	0 (0.0)

**Travel time to hospital, hours (*n*= 276)^b^**	
≤1	260 (94.2)
>1	16 (5.8)

**Travel time to GP, minutes (*n*= 296)^c^**	
≤10	187 (63.2)
>10	109 (36.8)

**Out-patient appointments in last 6 months (*n*= 272)**	
0–2	225 (82.7)
≥3	47 (17.3)

a *Unless otherwise stated.*

b *For travel to hospital: 207 (75.0%) travelled by car, 12 (4.3%) walked, 25 (9.1%) by taxi, and 29 (10.5%) by bus; answer not provided,* n *= 3 (1.1%).*

c *The majority (*n *= 191, 64.5%) reported travelling to their GP by car, with 72 (24.3%), 13 (4.4%), and 11 (3.7%) reporting walking, taxi, and bus, respectively as their means of travel; answer not provided,* n *= 9 (3.0%).*

### Change in treatment burden

**Figure 2.**
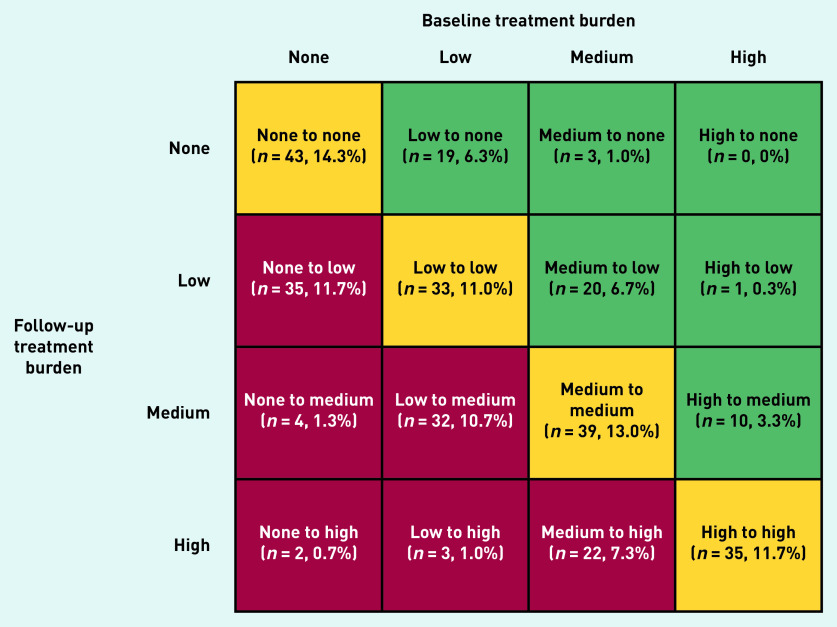
*Variation in treatment burden category status between baseline and follow-up.*

Overall, there was a mean (SD) increase of 2.6 (11.2) points in treatment burden global score at 2.5-year follow-up. In total, 151 people (50.3%) experienced a change in treatment burden category, with 32.7% ( *n* = 98) and 17.7% ( *n* = 53) experiencing an increase and decrease, respectively. Twenty-seven (9.0%) participants moved from a lower category to ‘high’ treatment burden (Figure 2).

Figure 2 presents a matrix of the dynamics of treatment burden in the study sample. A higher proportion of those experiencing an increase in treatment burden category were older (aged ≥75 years) and reported being ex-smokers, homeowners, with >3 LTCs, and prescribed >3 medications (although differences were not statistically significant). A lower proportion of those experiencing a decrease in treatment burden category were female and lived alone. A higher proportion experiencing a decrease in treatment burden category were employed and lived ≤10 minutes from their GP (although differences were not statistically significant). Supplementary Table S2 and Supplementary Figure S1 present characteristics of participants that experienced change in treatment burden category.

### Associations with change in treatment burden

Univariable regression models indicated that high treatment burden category (compared with no burden) at baseline was associated with a decrease in treatment burden global score (as a continuous outcome measure), β −4.85 (95% confidence interval [CI] = −8.80 to −0.91, where β is the effect of one unit change in a predictor on a one unit increase in global score change) (Table 2). Having >5 LTCs (β 5.89, 95% CI = 1.98 to 9.80) and living >10 minutes from the GP (β 3.89, 95% CI = 1.27 to 6.52) were associated with an increase in treatment burden.

**Table 2.** Associations with change in treatment burden global score

**Characteristic**	**Univariable**			**Multivariable^a^**		
	
	**β^b^**	**95% CI**	***P*-value^c^**	**β^b^**	**95% CI**	***P*-value^c^**
**Age category, years (versus 55–64)**						
65–74	0.197	–4.13 to 4.52	0.93	–1.08	–5.35 to 3.19	0.620
75–84	2.461	–1.92 to 6.84	0.27	1.70	–2.65 to 6.05	0.445
≥85	4.27	–1.11 to 9.66	0.12	3.17	–2.33 to 8.68	0.258

**Sex, female (versus male)**	–0.61	–3.14 to 1.91	0.64	–0.29	–2.87 to 2.29	0.825

**Marital status (versus married)**						
Single	–2.32	–9.00 to 4.36	0.44	–2.30	–8.75 to 4.14	0.484
Divorced or dissolved partnership	1.67	–2.53 to 5.86	0.50	2.07	–2.07 to 6.22	0.327
Widowed	0.12	–2.97 to 3.21	0.94	–1.87	–5.22 to 1.48	0.273

**Lives alone (versus with others)**	–0.93	–3.66 to 1.80	0.50	—	—	—

**Non-homeowner (versus homeowner)**	–2.16	–5.57 to 1.26	0.22	—	—	—

**Employment status (versus employed)**						
Unemployed	–2.29	–12.15 to 7.56	0.65	—	—	—
Retired	2.06	–2.58 to 6.69	0.39	—	—	—
Other	–0.48	–10.35 to 9.40	0.92	—	—	—

**Smoking status (versus never smoked)**						
Ex-smoker	0.94	–1.61 to 3.49	0.47	—	—	—
Current smoker	–4.35	–10.64 to 1.94	0.18	—	—	—

>**5 long-term conditions (versus** ≤**5)**	5.89	1.98 to 9.80	**0.003**	8.26	4.20 to 12.32	**<0.001**

≥**7 medications prescribed (versus** <**7)**	2.48	–0.03 to 4.98	0.05	—	—	—

**Limited health literacy (versus not limited)**	0.46	–3.40 to 4.31	0.82	—	—	—

**Some financial difficulty (versus none)**	–0.22	–5.68 to 5.23	0.94	—	—	—

≥**1 hour to hospital (versus**>**1 hour)**	1.22	–4.61 to 7.04	0.68	—	—	—

>**10 minutes travel time to GP (versus**≤**10 minutes)**	3.89	1.27 to 6.52	**0.004**	3.88	1.32 to 6.43	**0.003**

≥**3 out-patient appointments past 6 months (versus** <**3)**	–2.55	–5.90 to 0.79	0.13	—	—	—

≥**3 GP appointments in past 6 months (versus** <**3)**	–1.44	–3.99 to 1.10	0.27	—	—	—

**Baseline MTBQ category (versus no burden)**						
Low	–0.82	–4.11 to 2.46	0.62	–0.67	–3.91 to 2.56	0.68
Medium	–0.43	–3.75 to 2.88	0.80	–1.34	–4.62 to 1.92	0.42
High	–4.85	–8.80 to −0.91	**0.016**	–7.00	–11.04 to −2.96	**0.001**

a *Adjusting for age, sex, marital status, travel time to GP, number of long-term conditions, and baseline treatment burden category.*

b *β is the effect of one unit change in predictor on a one unit increase in global score change.*

c *Results in bold are significant at the P < 0.05 level. MTBQ = Multimorbidity Treatment Burden Questionnaire.*

Associations with change in treatment burden remained significant after adjusting for age, sex, marital status, time to GP, number of LTCs, and baseline treatment burden category: β −7.00 (95% CI = −11.04 to −2.96), β 8.26 (95% CI = 4.20 to 12.32), and β 3.88 (95% CI = 1.32 to 6.43) for high treatment burden category, number of LTCs, and time to GP, respectively (Table 2). The remaining variables were not significantly associated with change in treatment burden. The only significant univariable association with change in treatment burden category was that those in the baseline medium treatment burden MTBQ category were less likely to increase category (versus those in the no burden category, odds ratio 0.37, 95% CI = 0.19 to 0.71, *P* = 0.003) (see Supplementary Table S3).

Additional linear mixed regression models indicated higher travel time to GP was more strongly associated with an increase in treatment burden value for participants with limited health literacy compared with those with higher literacy; mean difference: β 10.41 (95% CI = 2.85 to 17.96) and β 9.59 (95% CI = 2.17 to 17.00), in unadjusted and adjusted models, respectively (data not shown).

### Single-item measure

A response of ‘yes’ to the single-item measure had a sensitivity of 55.7%, specificity of 92.4%, positive predictive value of 65.4%, and negative predictive value of 89.0%. The positive likelihood ratio was 7.34 (95% CI = 4.46 to 12.07) and the negative likelihood ratio was 0.48 (95% CI = 0.36 to 0.64). The AUC was 0.74 (95% CI = 0.68 to 0.81) (data not shown).

The Checklist for Reporting of Survey Studies (CROSS) was completed.^23^

## DISCUSSION

### Summary

This longitudinal study identified that half the sample of older people with multimorbidity experienced a change in treatment burden category in 2.5 years with this increasing for 33% and decreasing for 18%. Having >5 LTCs and greater travel time to the patient’s GP were statistically significantly associated with an increase in treatment burden global score (but not treatment burden category). With regard to travel time to GP, this was particularly the case for participants with limited health literacy. The authors’ single-item treatment burden measure performed moderately, suggesting further development of this measure is still needed.

### Strengths and limitations

To the authors’ knowledge, this is the first quantitative population-level study to describe change in treatment burden over time in the UK. Key strengths were the use of a validated measure of treatment burden, minimal non-response bias, and the inclusion of geographically dispersed and socioeconomically diverse GP practices, allowing a range of participants to be included.^17^ Furthermore, patient-level follow-up will have reduced unobserved confounding.

However, there were important limitations. First, the follow-up survey was conducted in the wake of the COVID-19 pandemic. This may have resulted in an underestimate in treatment burden change as people may not have been attending as many appointments, either in-person or virtually, or there may have been changes in practice (such as more telephone consultations) that influenced treatment burden. During the pandemic GP consultations in England changed dramatically, with face- to-face appointments decreasing initially (February to April 2020) then increasing substantially by August 2021, with telephone consultations almost trebling.^24^ For some, the lack of social support or reduced contact with a health professional may have increased treatment burden.^25^^–^30 Surveys were mailed out at a point of minimal COVID-19 restrictions to try and minimise these effects. Second, although exceeding the minimum target sample size, the study may have been underpowered to assess some associations with treatment burden category change and the clinical significance of a single- point change in MTBQ global score has not been well established. Third, associations with treatment burden are based on self-reported survey data that may be subject to recall bias and some participants may have interpreted questions differently. Fourth, it is not possible to make any causal inferences of the associations presented here. Finally, the Dorset population has relatively low levels of deprivation and low ethnic diversity, so findings may not be widely generalisable. Participants who were ineligible to receive the survey (that is, those excluded by practices) may have had higher treatment burden than those included, resulting in underestimation of high treatment burden.

### Comparison with existing literature

The overall increase in treatment burden may be a result of changes in individual factors such as an increased number of LTCs and medications at follow-up.^25^ COVID-19 pandemic factors such as lack of continuity of care, inadequate information, and difficulty accessing health care may also have contributed to this overall increase.^25^^–^30 The finding in the current study of a positive association between travel time to GP and an increase in treatment burden is consistent with findings from qualitative studies on the time burden associated with travel to health care.^31^^,^^32^ This association was moderated by health literacy, in line with studies suggesting health literacy may be protective against experiencing high treatment burden.^3^^,^^33^ Unlike previous cross-sectional treatment burden studies, the current study found no association between sociodemographic factors and change in treatment burden.^17^^,^^34^

A recent prospective study in the US evaluated treatment burden trajectories among 396 people (aged ≥20 years) with multimorbidity.^35^ The study measured treatment burden at four time points over 2 years and identified differing patterns of change between treatment burden ‘workload’ and ‘impact’. Workload trajectory was broadly represented in two groups — persistently high and persistently low, whereas impact had three patterns — consistently high, increasing, and consistently low. Consistently high workload was associated with lower health literacy, lower self-efficacy, and higher interpersonal challenges with others, whereas consistently high impact was associated with more mentally unhealthy days, lower health literacy, and higher interpersonal challenges with others. Increasing impact was associated with more physically unhealthy days and higher interpersonal challenges with others.^35^ The current study was only able to assess two time points, had an older population, and considered a different range of LTCs, and, in contrast to their findings, identified higher number of LTCs as independently associated with increasing treatment burden, and assessed aspects of access to health care, of which distance from a GP was independently associated. Some important differences in these studies are summarised in Supplementary Table S4.

The authors’ revised single-item measure needs further development, and a single question may not adequately capture the different components of treatment burden. It may be more prone to sociopsychological biases and random error, making it less stable and precise.^36^

### Implications for research and practice

This study emphasised the further need to consider the factors influencing burden and mitigate them where possible. Factors such as improving access to primary care, particularly for those living further away from services, may reduce treatment burden. This may include further consideration about modes of health service delivery to specifically meet the needs of those patients more likely to feel overburdened.^37^ Better education or more simplified information may be needed to allow patients to make more adequate choices regarding their health care. The finding in the current study that greater travel time to GP services is associated with an increase in treatment burden may be somewhat unexpected given more appointments were delivered virtually during the COVID-19 pandemic than prior to the pandemic.^24^^,^^38^ Although there would have been some face-to-face appointments, this finding may also indicate that virtual consultations increase burden for some patients.^29^^,^^39^ Larger population-level studies are needed to confirm these findings. The impact of health system changes was likely affected by the COVID-19 pandemic and it may not be possible to unpick these issues. Further qualitative research is needed to better understand patients’ views of treatment burden post-pandemic and the practical health service barriers and facilitators to managing multimorbidity. Development of a brief measure of treatment burden consisting of more than one item may be needed for use in clinical practice.

## Data

Data may be available on request from the authors.
